# Monitoring changing patterns in HER2 addiction by liquid biopsy in advanced breast cancer patients

**DOI:** 10.1186/s13046-024-03105-9

**Published:** 2024-06-29

**Authors:** Elena Giordani, Matteo Allegretti, Alberto Sinibaldi, Francesco Michelotti, Gianluigi Ferretti, Elena Ricciardi, Giovanna Ziccheddu, Fabio Valenti, Simona Di Martino, Cristiana Ercolani, Diana Giannarelli, Grazia Arpino, Stefania Gori, Claudia Omarini, Alberto Zambelli, Emilio Bria, Ida Paris, Simonetta Buglioni, Patrizio Giacomini, Alessandra Fabi

**Affiliations:** 1 Translational Oncology Research, IRCCS Regina Elena National Cancer Institute, Rome, Italy; 2 Department of Basic and Applied Sciences for Engineering, SAPIENZA University of Rome, Rome, Italy; 3 Division of Medical Oncology 1, IRCCS Regina Elena National Cancer Institute, Rome, Italy; 4 UOC Anatomy Pathology and Biobank, IRCCS Regina Elena National Cancer Institute, Istituti Fisioterapici Ospitalieri, Rome, Italy; 5 Pathology Unit, IRCCS Regina Elena National Cancer Institute, Rome, Italy; 6 Facility of Epidemiology and Biostatistics, Fondazione Policlinico Universitario Agostino Gemelli IRCCS, Rome, Italy; 7 Oncology Division, Department of Clinical Medicine and Surgery, University Federico II, Naples, Italy; 8 Medical Oncology, IRCCS-Sacro Cuore Don Calabria Hospital, Negrar di Valpolicella, Verona, Italy; 9 Division of Medical Oncology, Department of Oncology and Hematology, University Hospital of Modena, Modena, Italy; 10 Oncology Unit, ASST Papa Giovanni XXIII, Bergamo, Italy; 11 Medical Oncology, Università Cattolica del Sacro Cuore, Rome, Italy; 12 Comprehensive Cancer Center, Fondazione Policlinico Universitario Agostino Gemelli, IRCCS, Rome, Italy; 13 Department of Woman and Child Health, Fondazione Policlinico Universitario Agostino Gemelli IRCCS, Rome, Italy; 14 Precision Medicine Unit in Senology, Fondazione Policlinico Universitario Agostino Gemelli IRCCS, Largo Agostino Gemelli, 8, 00168 Roma, Italy

**Keywords:** HER2-positive breast cancer, Trastuzumab emtansine (T-DM1), Liquid biopsy, Circulating cell-free DNA (cfDNA), Circulating soluble HER2 (sHER2)

## Abstract

**Background:**

During targeted treatment, HER2-positive breast cancers invariably lose HER2 DNA amplification. In contrast, and interestingly, HER2 proteins may be either lost or gained. To longitudinally and systematically appreciate complex/discordant changes in HER2 DNA/protein stoichiometry, HER2 DNA copy numbers and soluble blood proteins (aHER2/sHER2) were tested in parallel, non-invasively (by liquid biopsy), and in two-dimensions, hence HER2-2D.

**Methods:**

aHER2 and sHER2 were assessed by digital PCR and ELISA before and after standard-of-care treatment of advanced HER2-positive breast cancer patients (*n*=37) with the antibody-drug conjugate (ADC) Trastuzumab-emtansine (T-DM1).

**Results:**

As expected, aHER2 was invariably suppressed by T-DM1, but this loss was surprisingly mirrored by sHER2 gain, sometimes of considerable entity, in most (30/37; 81%) patients. This unorthodox split in HER2 oncogenic dosage was supported by reciprocal aHER2/sHER2 kinetics in two representative cases, and an immunohistochemistry-high status despite copy-number-neutrality in 4/5 available post-T-DM1 tumor re-biopsies from sHER2-gain patients. Moreover, sHER2 was preferentially released by dying breast cancer cell lines treated *in vitro* by T-DM1. Finally, sHER2 gain was associated with a longer PFS than sHER2 loss (mean PFS 282 vs 133 days, 95% CI [210-354] vs [56-209], log-rank test *p*=0.047), particularly when cases (*n*=11) developing circulating HER2-bypass alterations during T-DM1 treatment were excluded (mean PFS 349 vs 139 days, 95% CI [255-444] vs [45-232], log-rank test *p*=0.009).

**Conclusions:**

HER2 gain is adaptively selected in tumor tissues and recapitulated in blood by sHER2 gain. Possibly, an increased oncogenic dosage is beneficial to the tumor during anti-HER2 treatment with naked antibodies, but favorable to the host during treatment with a strongly cytotoxic ADC such as T-DM1. In the latter case, HER2-gain tumors may be kept transiently in check until alternative oncogenic drivers, revealed by liquid biopsy, bypass HER2. Whichever the interpretation, HER2-2D might help to tailor/prioritize anti-HER2 treatments, particularly ADCs active on aHER2-low/sHER2-low tumors.

**Trial registration:**

NCT05735392 retrospectively registered on January 31, 2023 https://www.clinicaltrials.gov/search?term=NCT05735392

**Supplementary Information:**

The online version contains supplementary material available at 10.1186/s13046-024-03105-9.

## Background

The Human Epidermal growth factor Receptor 2 (HER2) status is routinely assigned by a two-sided testing algorithm taking into account gene over-expression and amplification. As per international guidelines, HER2 protein levels are quantified by immunohistochemistry (IHC) followed (when appropriate) by cytogenetic assessment of Deoxyribonucleic Acid (DNA) copy numbers. A positive HER2 status at a single time point, typically in breast cancer tissue obtained at diagnosis, is the minimum requirement to assign anti-HER2 therapy [1]. However, this is nothing more than a pragmatic and clinically useful simplification, because HER2 DNA copy numbers and protein levels change extensively during treatment, the latter in many different ways.

Amplified HER2 (aHER2) is invariably lost regardless of the clinical setting (early or metastatic disease) and testing method, e.g. whether assessed on tumor tissue DNA (tDNA) [2–4] or circulating cell-free DNA (cfDNA) [5–8]. In sharp contrast, HER2 proteins may instead be gained, as noted in circulating tumor cells (CTCs) [9–11] and in blood, where they are released in soluble form (sHER2). For instance, sHER2 gain above the normal cut-off threshold of 15 ng/ml, approved long time ago by the Food and Drug Administration (FDA) [12], was proposed to mark progression during anti-HER2 treatment [12–15]. However, as also noted in a recent meta-analysis [15], most of the >12,000 patients with published sHER2 data are from early clinical trials enforcing homogeneous enrolment criteria and receiving naked antibody treatment. No sHER2 validation studies were carried out with Antibody-Drug Conjugates (ADC), possibly because these were introduced later in the clinics. Therefore, sHER2 levels and thresholds are hardly applicable to real-world populations, that presently widely differ in tumor burden and exposure to diverse classes of anti-HER2 agents. Possibly for these reasons, sHER2 testing and threshold have never gained widespread acceptance. As a result, re-assessment of the HER2 status, sometimes necessary for routine patient management, still largely relies on invasive tumor re-biopsy rather than sHER2.

Another limitation of the available studies dealing with HER2 status re-assessment is that aHER2 and/or sHER2 were not systematically investigated across multiple lines of therapy. Relevant to this point, aHER2 (but not sHER2) was monitored by combined tDNA/cfDNA testing in our own LiqBreasTrack study. In LiqBreasTrack, the expected aHER2 loss was indeed detected. It was slow during early treatment lines with the naked therapeutic antibodies Trastuzumab, and Trastuzumab plus Pertuzumab (T and T+P), and then it became rapid and reached completion in most patients within months of further treatment with Trastuzumab-emtansine (T-DM1) [16]. This was of interest to us, because T-DM1 requires HER2 over-expression to induce optimal clinical response [17, 18]. Then, given the known link between HER2 amplification and over-expression, patients displaying residual blood aHER2, followed by rapid T-DM1-mediated suppression, were expected to host HER2-high tumors, and have a favorable outcome. However, outcome was not significantly different in these patients [16], questioning the significance of circulating aHER2, at least *per se*.

On this basis, it was hypothesized that aHER2/sHER2 testing, simultaneous and longitudinal, would provide a more comprehensive description of adaptive changes in HER2 oncogenic dosages. To test this hypothesis, a non-invasive liquid biopsy proxy was herein developed, validated, and applied to advanced breast cancer patients. By measuring circulating aHER2 [5–8, 16] by dPCR, and sHER2 [12] by a sandwich ELISA, this assay recapitulates two-dimensional HER2 status assessment in tumor tissues, hence HER2-2D. Since it is non-invasive, HER2-2D could be systematically applied in the context of two prospective studies enrolling patients treated with T-DM1: the cited LiqBreasTrack study (*n*=20), and the multicenter LiqERBcept/GIM21 (*Gruppo Italiano Mammella*) trial (*n*=17), the latter currently ongoing.

Results from these 37 patients confirm a generalized aHER2 loss under T-DM1 pressure, but surprisingly reveal a discordant sHER2 gain (e.g. HER2 split). Moreover, sHER2 gain is not associated with progression and poor outcome, as observed during treatment with naked antibodies [15], but with a prolonged clinical response to T-DM1. Interpretations are proposed to reconcile these apparently contradictory HER2 stoichiometries and outcome associations. It is also suggested that HER2-2D may aid in therapeutic assignments.

## Methods

### Study design and patients

HER2-2D includes prospectively enrolled patients from the cohort, single-arm, minimally interventional (blood drawing) LiqBreasTrack and LiqERBcept (NCT05735392) studies (*n*=20 and 17, respectively). Blood obtained prior to T-DM1 treatment was available from 41 patients. These specimens were used in a preliminary assay validation phase. Thirty-seven of these 41 patients had paired blood samples available, obtained at baseline and progression during T-DM1 treatment. These were used for HER2-2D testing. The features of these 37 patients are summarized in Table 1.

**Table 1:** Demographics and clinical pathological features of patients in the HER2-2D cohort

**Characteristics**	**N**
Age, years (range)	56.8 (33-87)
Previous lines of therapy^a^	
0	1
1	14
2	5
3	2
Patients treated with naked therapeutic antibodies	
Trastuzumab	3
Trastuzumab + Chemotherapy	7
Trastuzumab + Pertuzumab	2
Trastuzumab + Pertuzumab + Chemotherapy	20
Dominant Metastatic sites	
Bone	18
Lymph Node	18
Lung	13
Liver	11
Breast	9
Pleura	9
Brain	7
Soft tissues	4
Number of metastatic sites per patient	
1	8
2	14
≥ 3	15

^a^Including: Lapatinib plus Capecitabine, Trastuzumab plus Vinorelbine, and Trastuzumab plus Carboplatin

The primary aims of LiqBreasTrack and LiqERBcept were to enumerate genomic alterations in cfDNA prior to and following T-DM1 treatment, and to correlate their appearance in blood with medical imaging data, respectively. Running HER2-2D was a secondary aim and pre-specified analysis of LiqERBcept only. Sample size was calculated to allow monitoring a sufficient number of alterations in cfDNA to meet the primary aim. There was no pre-specified sample size for HER2-2D analysis. None of the 37 patients withdrew or was lost to follow-up. T-DM1 was administered at 3.6 mg/kg i.v. every 21 days until progression, unacceptable toxicity or patient refusal, as per Standard of Care (SoC) in advanced HER2-positive breast cancer at the time (years 2018-2021) of recruitment. Inclusion criteria: (a) >18 year old; (b) ventricular ejection fraction >50%; (c) Eastern Cooperative Group (ECOG) performance 0 or 1; (d) HER2-positive advanced breast cancer progressing from previous treatment with Trastuzumab, Trastuzumab/Pertuzumab, with or without taxanes (any number of previous therapy lines for LiqBreasTrack, mandatory one line only for LiqERBcept); (e) availability of primary tumor tissue. Exclusion criteria: (a) previous treatment with T-DM1; (b) symptomatic brain metastases at enrolment; (c) enrolment in clinical trials during the previous 4 months; (d) heart failure or cardiac infarction during the past 6 months.

### Blood drawing

Baseline (T_0_) blood was drawn at progression from previous treatment, right before the first T-DM1 administration. A second blood sample was obtained at T-DM1 progression (T_p_). Blood was processed by the so-called 2-spin protocol [19], and single-use aliquoted at -80°C. Additional blood from patients with glioblastoma (*n*=4), thyroid cancer (*n*=4), and healthy donors (*n*=8) was from the Regina Elena institutional Biobank.

### HER2 dPCR assay

cfDNA was purified from 4 ml of plasma by the QIAmp circulating nucleic acid kit (Qiagen), and eluted in 30 μl, of which 6.5 μl (corresponding to approximately 0.86 ml of plasma) were assessed in the chip-based QuantStudio™ 3D Digital PCR System (Life Technologies). aHER2 was computed as the DNA copy number ratio (test vs control gene) between HER2 and the Elongation Factor TU GTP binding Domain 2 (EFTUD2). A dPCR normal blood threshold of 1.25 (HER2/EFTUD2 copy number) was validated in the above study and independently confirmed by other groups including ourselves [5–7, 16]. For dPCR primers see supplementals.

### Quantitative HER2 ELISA

HER2 levels in tissues and blood (sHER2) were measured (OD_450_ nM) as the average ± Standard Deviation (SD) of triplicates using a two-antibody sandwich Human HER2 DuoSet assay (R&D System, MN, USA), following optimization of capture and detection antibodies (5.0 and 0.25 μg/ml respectively) by interpolation on a standard curve (two-fold dilutions from 3.5 to 0.054 μg/ml of a recombinant human HER2/Fc Chimera). Each ELISA run was normalized relative to the standard curve run in parallel. Data were reduced by a four-parameter logistic curve fit using GraphPad Prism v9.0. The lower limit of quantification (1 ng/ml) was the lowest nonzero concentration level which could be accurately and reproducibly quantitated. Optimal assay inputs per well were as follows: cell and tissue lysates 0.5 μg; tissue culture supernatants and plasma 1μl. In some elaborations, the FDA normal blood threshold was applied of 15 ng/ml [12, 13].

### Tumor tissues and cells

Archival tissues (formalin-fixed, paraffin-embedded) were obtained from primary and metastatic HER2-positive breast cancers prior to T-DM1 administration, and occasionally from accessible metastatic sites at progression (re-biopsy), as described [16]. Immunohistochemistry (IHC) was carried out by staining with the polyclonal antibody A0485 (Dako, Denmakk) to HER2 at 2.0 μg/ml, following antigen retrieval at pH 6 in citrate buffer. Immunoreactions were revealed by Bond Polymer Refine Detection in an automated autostainer (Bond III, Leica Biosystems), and acquired by an Aperio AT2 (Leica) instrument in a full CE-IVD environment/workflow. HER2 was scored as per international guidelines. For additional details on tissue lysates see Supplementary Methods. T-DM1 was obtained from injectable preparations for human infusion (Roche Pharmaceuticals), and added to adherent cell cultures growing under standard conditions. At the indicated times, cells were detached, counted, and lysed in CST lysis buffer (Thermo Fisher Scientific) as described in Supplemental Methods. Equal amounts of cell lysates (BCA-normalized for protein content) were resolved by SDS-PAGE, and Western-blotted onto nitrocellulose filters for binding to anti HER2 and Heat Shock protein 70 (HSP) antibodies (both from Cell Signaling Technology, Danver, MA). Cell lines and other antibodies are described in more details in Supplemental Materials.

### Statistics

Progression-free survival (PFS) was calculated from the first T-DM1 administration to progression or death, observed in all 37 patients. PFS curves were estimated with the Kaplan-Meier method and compared with the log-rank test. Association between quantitative variables were assessed by R-squared. Data were elaborated by GraphPAD Prism v9.0 (RRID:SCR_002798; GraphPad Software, CA, USA), as described [16]. Two-sided *p* values <0.05 were considered statistically significant.

## Results

### aHER2 and sHER2 testing: analytical validation, assay optimization and specificity

In preliminary experiments, the dPCR and the ELISA sandwich assays were individually validated, and then combined into HER2-2D. Testing of widely available cell lines with known levels of HER2 DNA copy number and (over)expression demonstrated that the two assays are accurate and detect the expected correlation between HER2 amplification and over-expression (Fig. S1a-d).

**Fig. 1:**
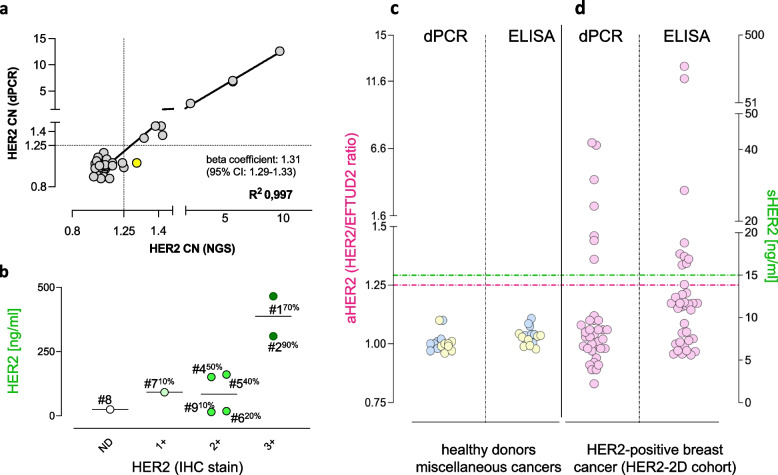
Validation of dPCR and sandwich ELISA in breast cancer blood and tissues. **a** Linear regression analysis of paired HER2 DNA copy number values orthogonally assessed by dPCR (normalized by reference to EFTUD2), and NGS (normalized by reference to a multi-gene baseline) in 41 cfDNA samples obtained prior to T-DM1 administration. Regression, 95% confidence intervals (CI), and beta coefficient are shown. Dotted lines define the 1.25 threshold of HER2 amplification. A single outlier (NGS-amplified/dPCR-neutral) is depicted in yellow. **b** ELISA testing of lysates from breast carcinoma tissues of untreated patients at the optimal protein input of 0.5 μg/well. Tissues are sorted by HER2 IHC staining intensity (in abscissae) and color-coded. ND=not detectable/bare staining traces. Per cent tumor fraction is noted for each (#1 to #9) tissue. **c** and **d** dPCR (HER2/EFTUD2 ratios) and sandwich ELISA testing of blood from healthy donors (light blue), from patients with tumors other than HER2-positive breast cancer (light yellow), and patients with HER2-positive breast cancer (light red) prior to (T_0_) T-DM1 administration. Dotted lines: blood aHER2 and sHER2 thresholds, color-coded

Further aHER2 and sHER2 validation was carried out on clinical specimens. cfDNAs (*n*=41) obtained prior to T-DM1 treatment from HER2-positive patients enrolled in the LiqBreasTrack and LiqERBcept studies were orthogonally tested by the HER2/EFTUD2 dPCR assay and a targeted NGS panel (Oncomine Pan-Cancer Cell-Free Assay, Thermofisher). The former measures HER2 DNA copy numbers relative to a control gene (EFTUD2), shown in a previous study [5] to be superior to any other gene comparator on chromosome 17, including pericentromeric normalizers such as the CEP17 gene used in Chromogenic In Situ Hybridization (CISH). The latter measures DNA copy numbers of 12 genes (including HER2) relative to the average DNA copy numbers of all genes in the panel. Despite extremely different normalization approaches, regression analysis of DNA copy numbers demonstrated (Fig. 1a) strong linear association (R^2^ >0.99) and concordance (beta coefficient = 1.31) between the two assays, with narrow confidence intervals (95% CI: 1.29-1.33). Likewise, when the pre-defined [5, 7] 1.25 aHER2 amplification threshold was applied to both assays (dotted lines), amplification was concordantly assigned by dPCR and NGS in 40/41 cases (98% approximately), resulting in a single outlier (yellow). Thus, aHER2 levels and a normal/non-amplified status were concordantly defined by independent assays.

The ELISA sandwich assay was similarly optimized and validated on clinical specimens. To this end, breast carcinoma tissues were obtained representative of different molecular subtypes (HER2-positive and HER2-negative), as assessed by Immunohistochemistry (IHC) and CISH using CEBP17 as an internal normalizer. All specimens were obtained at diagnosis from untreated patients, and tested as per international ASCO-CAP diagnostic guidelines [1]. Protein lysates were then prepared from frozen tissue aliquots, and serially diluted to identify the lysate input resulting in optimal discrimination among widely different HER2 levels in tissues (Fig. S1e). At this predetermined optimal input, HER2 3+ tissues (*n*=2) were highest, whereas IHC-negative/1+/2+ tissues were in the low ELISA binding bracket, and were poorly resolved (Fig. 1b). Inspection of hematoxylin/eosin sections revealed that the lowest ELISA values had been detected in tissue samples with low HER2 and/or low tumor cellularity (noted in Fig. 1b), as expected. Nevertheless, entering ELISA and HER2 DNA copy number values (assessed in genomic DNAs from the same frozen tissues by dPCR) into the HER2-2D plot resulted in a significant linear regression (Fig. S1f). In summary, sandwich ELISA discriminated HER2-positive from HER2-negative/low breast cancer tissues, and detected a correlation between HER2 amplification and over-expression in tissues from untreated breast cancer patients.

As a final validation step, both aHER2 and sHER2 were assessed in blood. Following the identification of the optimal plasma ELISA input (Fig. S1g), cfDNA and plasma were tested from 3 separate experimental groups: healthy donors (*n*= 8), patients bearing miscellaneous tumors that rarely host [20] HER2 amplification/over-expression (*n*=8), and HER2-positive advanced breast cancer patients from the LiqBreasTrack and LiqERBcept studies (*n*=37) at the time of progression from the anti-HER2 therapy line administered immediately before T-DM1 (see Table 1). As expected, both aHER2 and sHER2 were below their respective normal thresholds in healthy donors and patients bearing miscellaneous tumors (Fig. 1c), whereas either or both were above threshold in a minority (7 and 9 of 37, e.g. 19% and 24% respectively) of pre-treated HER2-positive breast cancer patients (Fig. 1d), as described for aHER2 [16]. Therefore, dPCR and ELISA are specific, detect aHER2 and sHER2 over wide concentration ranges, and both resolve HER2-positives from HER2-negatives in blood, justifying their use to accurately monitor patients receiving specific anti-HER2 treatments.

### aHER2 and sHER2 in patients treated by Trastuzumab and Trastuzumab plus Pertuzumab

Of 37 patients with matched T_0_-T_p_ samples in the HER2-2D cohort, 32 had received Trastuzumab (T) alone, or Trastuzumab plus Pertuzumab (T+P) for metastatic disease (Table 1) prior to T-DM1 treatment. Interestingly, at this time aHER2 and sHER2 were coordinated in all patients from the T group and most (77%) patients from the T+P groups (Fig. S2a and S2b). Thus, only minor aHER2/sHER2 dis-coordination was seen at baseline, prior to T-DM1 treatment.

### aHER2 and sHER2 during T-DM1 treatment

**Fig. 2:**
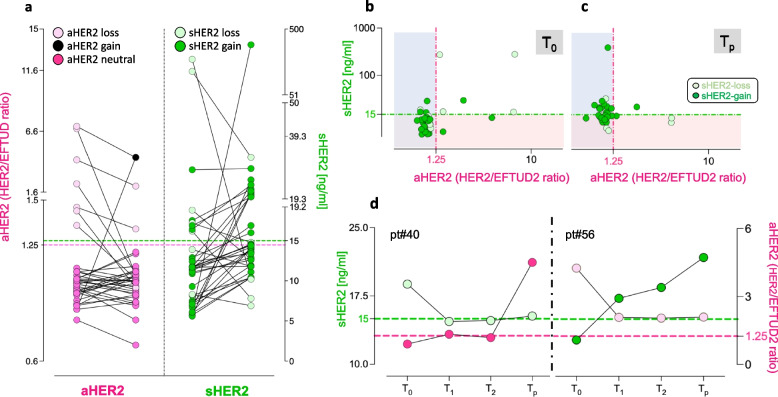
Changes in aHER2 and sHER2 in T-DM1-treated patients. **a** Baseline-progression (T_0_-T_p_) comparison of aHER2 and sHER2 by 1-d dPCR and ELISA plots in the entire cohort of 37 HER2-2D patients. Dots corresponding to five distinct aHER2 and sHER2 phenotypes are color-coded. aHER2 and sHER2 thresholds: dotted lines, color-coded. **b** HER2-2D plot of the same data. sHER2 gain, losses and thresholds are color-coded as in panel (**a**). **c** aHER2 and sHER2 time courses in two representative patients. Color coding as above

Next, aHER2 and sHER2 were tested in the 37 T-DM1-treated patients from the complete HER2-2D cohort, which includes the 32 patients previously treated with T and T+P. aHER2 and sHER2 were compared between baseline and progression (T_0_ vs T_p_), e.g. before the first T-DM1 administration (which coincides with progression from previous treatments), and after the last T-DM1 infusion. One-dimensional aHER2 plots (Fig. 2a, left) revealed three distinct patterns: loss in 7 patients (19%; dots in light red); persistence of a aHER2-neutral, below-threshold blood status (mean 1,01 ± 0,09) in 29 cases (78%; dots in dark red); and gain in one patient only (3%, black dot), although of considerable entity (from HER2-neutral in T_0_ to approximately 6 copies in T_p_). It may be concluded that aHER2 is either lost or persistently neutral in blood from most (36/37; 97%) T-DM1-treated patients.

Unlike aHER2 loss, sHER2 loss (Fig. 2a, right) was rare (7/37 cases, 19%, dots light green). The dominant phenotype, was a surprising sHER2 gain (30/37 cases, 81%; dots in dark green), almost invariably of considerable magnitude (median 1.45-fold; ranges 1.1 to 64.4)

For improved, patient-by-patient visualization of opposite trends, paired aHER2 and sHER2 values were displayed in two dimensions. These HER2-2D plots revealed (Fig. 2b vs 2c, T_0_ vs T_p_) a drastic depletion in aHER2/sHER2 double-positives associated with an upward sHER2 shift (dark green dots) particularly evident in the left-side (aHER2-negative) quadrants. Thus, the canonical HER2 amplification/over-expression linkage, still rather conserved after naked antibody treatment (T and T+P), was completely disrupted by T-DM1, mainly due to unorthodox, opposing aHER2 and sHER2 trends, e.g. frequent aHER2 loss or persistent neutrality vs frequent sHER2-gain. This phenotype is dubbed HER2 split hitherto.

### aHER2 and sHER2: time-course of HER2 split

Four longitudinal blood drawings were available from two patients representative of the frequent sHER2 gain (pt#56), and the rare sHER2-loss (pt#40) phenotypes, the latter remarkably associated with the above-noted, unusual aHER2 gain in this unique patient. Interestingly, aHER2/sHER2 trajectories were reciprocal (Fig. 2d), confirming that HER2 split results from opposing aHER2 and sHER2 trends.

### HER2 split in tumor re-biopsies

**Fig. 3:**
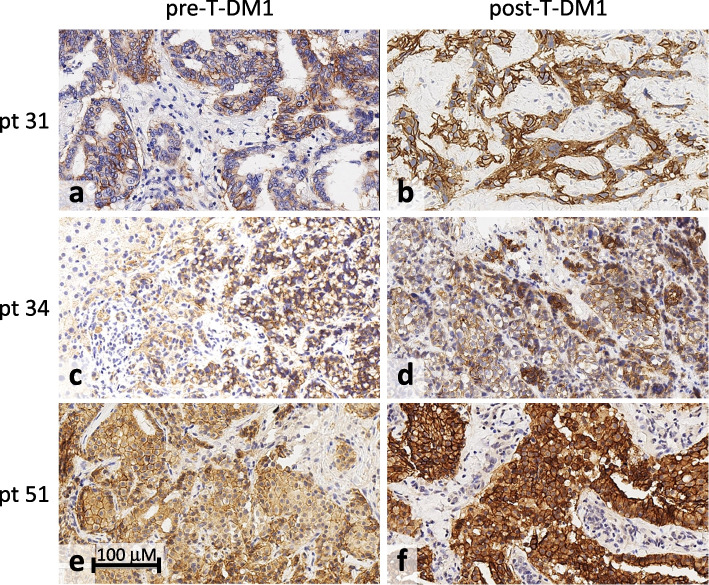
Immunohistochemistry of metastatic breast cancer lesions. HER2 IHC of metastatic lesions from HER2-positive breast cancers (as per assessment of the primary tumor at diagnosis), obtained from three patients before and after T-DM1 treatment, as indicated. Staining and scoring was as per international guidelines. Metastatic sites and IHC scores: (**a**) lung 2+; (**b**) lymph node 3+; (**c**) liver 1+; (**d**) liver 2+; (**e**) lymph node 2+; (**f**) pleura 3+

HER2 split was also investigated in tumor tissues. Re-biopsies could be safely obtained within 7 days of the T_p_ time point from skin and lymph node lesions of 5/30 patients displaying the frequent aHER2-loss/sHER2-gain phenotype. All 5 tumor tissues were confirmed to be HER2 DNA copy-number-neutral by dPCR. Yet, 4 of them displayed a strong homogeneous 3^+^ HER2 immunohistochemical stain. From three of these patients paired biopsies were available obtained less than a year before the beginning of T-DM1 treatment and within a week after the last T-DM1 administration. All of them displayed detectable HER2 gains (Fig. 3). Therefore, HER2 split in blood appears to largely recapitulate an unorthodox HER2 DNA-neutral/HER2-protein-high status in tumor tissues.

### T-DM1 induces HER2 release from dying tumor cells

**Fig. 4:**
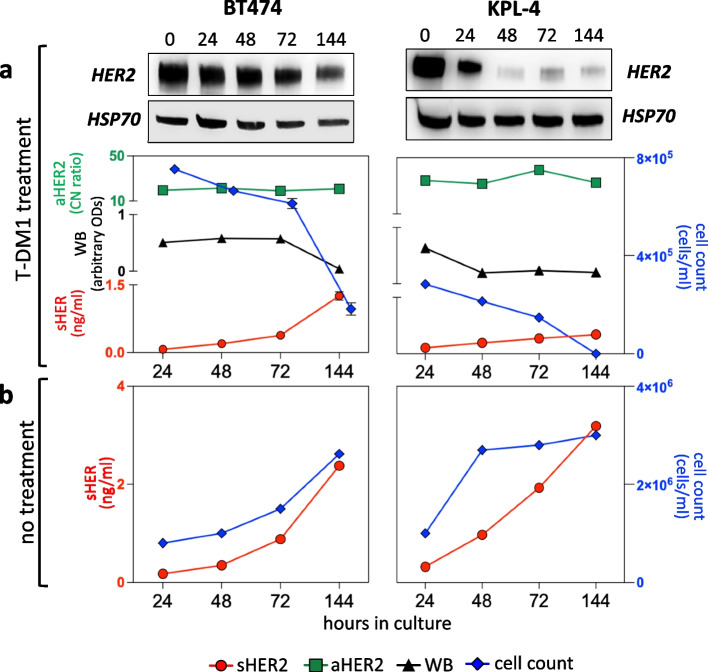
sHER2 release from HER2-positive breast cancer cells grown in the presence of T-DM1. Western blotting, aHER2, sHER2 and cell counts from the two indicated cell lines in a time-course (144h) experiment of T-DM1 treatment (1 μg/ml). From top to bottom: Western blot images, and graphical representation of the four measured variables (see bottom) including ODs of Western blotting scans by Image J (https://imagej.net). The different units in ordinates are also color-coded. Standard deviations of triplicate determinations are shown where they exceed the size of the markers

The BT474 and KPL-4 breast cancer cell lines were selected to investigate sHER2 gain. Both cells carry amplified and overexpressed HER2 (see Fig. S1a-d), but BT474 are susceptible whereas KPL-4 are resistant to Trastuzumab [21]. A short-term T-DM1 pulse (144h) was selected, with the limited aim to assess short-term T-DM1 effects. In preliminary experiments (not shown and see below), the highest tolerated T-DM1 concentration was identified (1 μg/ml) resulting in strong growth suppression, progressive and nearly complete cell killing, and clearly detectable effects on HER2 proteins, both intracellular and in the culture supernatant (sHER2). Treatment with T-DM1 at the pre-selected concentration resulted in similar patterns despite the considerable differences between cell lines. aHER2 changes were negligible. Cell numbers and cellular HER2 concordantly decreased and, interestingly, only sHER2 underwent a sharply divergent increase (Fig. 4a, Western blot images and densitometries). In contrast, cultures grown in parallel in the absence of T-DM1 displayed one log higher sHER2 levels, and parallel increases in cell growth and sHER2 levels, the latter evident even after fast-growing KPL4 cells reached plateau (Fig. 4b). In summary, sHER2 is mainly released by dying and growing cells in the presence and absence of T-DM1 respectively.

### sHER2 gain is associated with a favorable outcome

**Fig. 5.**
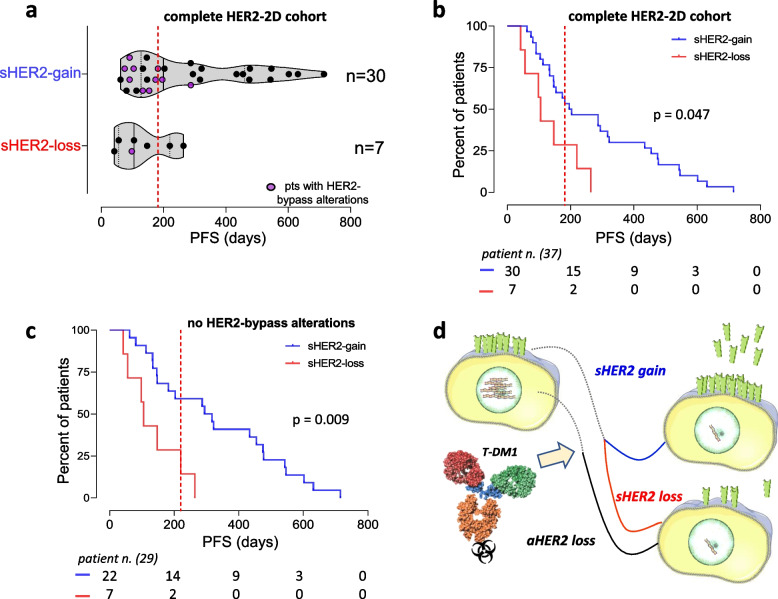
sHER2 and survival. PFS of T-DM1-treated HER2-2D patient subsets (sHER2-gain vs sHER2-loss). **a** Dot plots. Magenta dots: patients with circulating alterations other than aHER2 (bypass alterations). **b** Kaplan-Meier curves from the same dataset. **c** Kaplan-Meier curves after purging patients with HER2-bypass alterations. **d** Hypothetical HER2 split model: adaptive sHER2 gains and losses mirror HER2 changes in tumor tissues. Soft-wired sHER2/HER2 gain during T-DM1 treatment is viewed as a tumor countermeasure opposing the T-DM1-elusive effect of DNA copy number loss. (b and c) dotted red lines: median PFS (182 and 220 days, respectively)

Next, aHER2 and sHER2 were correlated with clinical outcome by Kaplan-Meier analysis. Progression-free survival (PFS) did not correlate with absolute T_0_ values of either aHER2 (not shown) or sHER2 (Fig. S3a/b; *p*=0.34, n.s.), but it was significantly longer in patients displaying sHER2-gain than in patients displaying sHER2-loss (Fig. 5a/b; mean PFS 282 vs 133 days, 95% CI [210-354] vs [56-209], log-rank test *p*=0.047). Therefore, the outcome of T-DM1 treatment correlates with dynamically assessed (T_0_ vs T_p_) sHER2 gain (which coincides with HER2 split), but not with absolute analyte measurements and static cut-off thresholds.

### HER2 split and bypass alterations

In LiqBreasTrack, several circulating alterations were found to undergo quantitative increases during T-DM1 treatment. Many of these were undetectable in the available tumor tissues, but could be detected *de novo* in blood. They were gain-of-function, mostly actionable, and involved breast cancer drivers other than HER2 [16], suggesting bypass of the HER2 oncogenic pathway. Interestingly, when assessed by NGS in the entire HER2-2D cohort, 9 distinct tumor-specific alterations were detected in blood, for a total of 11 mutational hits in 11 different patients, as follows: ESR1 p.D538G, p.Y537C, and p.Y537S; PIK3CA p.E545K, p.H1047R (in two patients); TP53 p.A276D (in two patients), p.C141Y, p.R213*, and p.S240R. Interestingly, most (10/11) hits occurred in early progressors from the favorable outcome sHER2-gain group, as shown by PFS values clustered below or right above median, and one hit was detected in the sHER2-loss group (Fig. 5a, magenta dots).

Unsurprisingly, when all 11 early relapsors were purged from the Kaplan-Meier model, the predictive ability of sHER2 gain improved (Fig. 5c; mean PFS 349 vs 139 days, 95% CI [255-444] vs [45-232], log-rank test *p*=0.009). It is concluded that sHER2 loss and sHER2 gain indicate poor and favorable response to T-DM1 respectively, and that HER2 bypass behaves as an independent variable predicting poor outcome despite the favorable influence of sHER2-gain.

## Discussion

Herein, the two-sided HER2 amplification/over-expression scheme of tissue diagnostics was copy-pasted into a two-dimensional (aHER2/sHER2) liquid biopsy assay called HER2-2D. Despite the limited sample size, the present study addresses in a novel way the long-vexing question of changes in HER2 expression/addiction during targeted therapy.

Testing by HER2-2D revealed that despite aHER2 was lost or persistently neutral in the blood of all but one of 37 T-DM1-treated patients, the dominant phenotype seen in most (30/37) of them at progression was sHER2 gain (Fig. 2). Interestingly, sHER2 gain was associated with long-lasting clinical responses to T-DM1 (Fig. 5). These findings are unprecedented and puzzling, but they are supported by several lines of evidence and considerations.

As to selective sHER2 gain without aHER2 gain (dubbed HER2 split herein), it contradicts the dogma of amplification-dependent HER2 over-expression. Although surprising, this finding is supported by three observations of ours: (a) HER2 levels were high and/or increased in 4/5 tested post-T-DM1 tumor re-biopsies, and all these were aHER2-neutral (Fig. 3); (b) although assessed in two patients only, aHER2 and sHER2 kinetics were reciprocal (Fig. 2d); (c) HER2 split was not seen at progression from Trastuzumab and was rare at progression from double Trastuzumab/Pertuzumab blockade (Fig. S2). Then, it may be concluded that HER2 split is an unprecedented phenotype originating in tumor tissues, recapitulated by liquid biopsy, and seen much more frequently upon treatment with T-DM1 than with naked antibodies.

As to sHER2 gain and favorable T-DM1 outcome, no association was evident when sHER2 was assessed as an absolute (above/below the FDA threshold) population metric (Fig. S3). Possibly, single-point, pre-treatment measurements relative to a defined threshold are confounded by marked patient-to-patient variation in tumor burden, number of metastatic foci (noted in Tab. 1), absolute blood sHER2 levels (Fig. 1b), and different tissue HER2 levels at both baseline and progression (Fig. 4). In agreement with this interpretation, sHER2 was associated with a favorable outcome only when dynamically assessed (baseline-vs-progression) as a patient-specific metric, irrespective of the FDA threshold. It is suggested that sHER2 dynamics captured by liquid biopsy are associated with defined outcomes because they most accurately infer a weighted average of HER2 protein gains occurring at tumor sites altogether. Thus, metrics based on the sHER2 threshold and sHER2 gains are alternative, and in our hands only the latter captures phenotypes associated with outcome.

One may then wonder why sHER2 gain, that is unfavorable in the Trastuzumab setting [15], as incidentally confirmed herein, is instead favorable in the T-DM1 setting. Further studies are clearly needed, but these contradictory/counterintuitive findings are readily reconciled by considering the profound differences between naked antibodies and ADCs. The former counteract copy number-dependent oncogenic signaling but, being marginally cytotoxic, cannot eliminate most targeted tumor variants. These are instead irreversibly wiped off, as shown previously [16] and confirmed herein, by a strongly cytotoxic ADC such as T-DM1. Accordingly, sHER2 release in the culture supernatant was proportional to cell growth in the absence of T-DM1, but became proportional to cell death in T-DM1-treated breast cancer cells, with an apparent relocation of HER2 from the intracellular compartment into the culture supernatant (Fig. 4). If sHER2 originates, at least in part, as a consequence of direct cytotoxic effects of T-DM1 on the tumor, its peculiar association with a favorable prognosis is more easily explained. However, we cannot exclude (and actually our results favor the possibility) that sHER2 is also released by live expanding cell subsets at sites of tumor progression. An important caveat is that short-term treatment in ‘closed’ *in vitro* models is too crude to mimic HER2 split in complex clinical setting. Accordingly, sHER2-high phenotypes are shared by cell cultures and tissue re-biopsies (Figs. 3 and 4), but only the latter lose aHER2 and acquire cellular HER2 expression, possibly through an unknown compensatory mechanism that cannot be seen in short-term cultures.

It is then postulated that aHER2 counterselection leaves aHER2-neutral breast cancers no alternative but re-gaining a sufficient oncogenic dosage through protein-only HER2 up-regulation. During naked antibody regimens this benefits the tumor, but with T-DM1 (and possibly other ADCs) it may also improve tumor targeting/elimination, ultimately keeping the drug-tumor balance in check, at least temporarily. This equilibrium is broken when HER2 addiction is eventually disrupted by other selective events, including HER2-bypass alterations (diagram in Fig. 5d), as suggested by the improved PFS-predictive ability of sHER2 gain when cases with circulating bypass alterations are disregarded (Fig. 5c).

## Conclusions

Whichever the preferred interpretation of HER2/sHER2 gain, the present study unequivocally identifies a small subset of fast progressors (most with below-median PFS) undergoing double and concerted aHER2/sHER2-loss. This phenotype, that might have been expected to be frequent, is instead rare. As shown by a considerable body of literature and also observed herein, these HER2/sHER2-low breast cancers are virtually T-DM1-untargetable. However, they may benefit from second-generation ADCs carrying cleavable payloads with bystander effect, such as Trastuzumab deruxtecan [22], and other ADCs like SYD985 [23]. Thus, HER2-2D may provide a quick composite biomarker for prompt therapeutic switch and ADC prioritization in patients at high-risk of developing tumor variants rapidly losing HER2 addiction.

### Supplementary Information

Supplementary Material 1.

## Data Availability

Data are available from the IRCCS Regina Elena National Cancer Institute website (www.ifo.it). Datasets, materials and laboratory protocols are available upon request.

## Abbreviations

ADC: Antibody-drug Conjugate
aHER2/sHER2: Amplified/soluble HER2 in blood
cfDNA: circulating cell-free DNA
DNA: Deoxyribonucleic Acid
CISH: Chromogenic In Situ Hybridization
dPCR: Digital PCR
EFTUD2: Elongation Factor TU GTP Binding Domain 2
FDA: Food and Drug Administration
HER2: Human Epidermal growth factor Receptor 2
NGS: next generation sequencing
PFS: Progression free Survival
T-DM1: Trastuzumab-emtansine
TTP: Time to Progression
