# Supplementary material for: Monitoring changing patterns in HER2 addiction by liquid biopsy in advanced breast cancer patients
Source: J Exp Clin Cancer Res. 2024 Jun 29;43:182. doi: 10.1186/s13046-024-03105-9 (PMC11218356; doi:10.1186/s13046-024-03105-9)
Supplement: Supplementary file 1 — Supplementary Material 1. [file 13046_2024_3105_MOESM1_ESM.pdf]

Monitoring changing patterns in HER2 addiction by liquid biopsy in advanced breast cancer patients

Elena Giordani[1], PhD; Matteo Allegretti[1], PhD; Alberto Sinibaldi[2], PhD; Francesco Michelotti[2], PhD; Gianluigi Ferretti[3], MD, PhD; Elena Ricciardi[1], BSc; Giovanna Ziccheddu[1], PhD; Fabio Valenti[1], PhD; Simona Di Martino[4], Cristiana Ercolani[4], Diana Giannarelli[5], PhD; Grazia Arpino[6], MD; Stefania Gori[7], MD; Claudia Omarini[8], MD; Alberto Zambelli[9], MD; Emilio Bria[10], MD; Ida Paris[11], MD; Simonetta Buglioni[4], PhD; Patrizio Giacomini[12,*], MD; and Alessandra Fabi[12], MD

[1] Translational Oncology Research, IRCSS Regina Elena National Cancer Institute, Rome, Italy

[2] Department of Basic and Applied Science for Engineering, Sapienza University, Rome, Italy

[3] Division of Medical Oncology 1, IRCSS Regina Elena National Cancer Institute, Rome, Italy

[4] Pathology Unit, IRCSS Regina Elena National Cancer Institute, Rome, Italy

[5] Facility of Epidemiology and Biostatistics, Fondazione Policlinico Universitario Agostino Gemelli IRCCS, Rome, Italy

[6] Oncology Division, Department of Clinical Medicine and Surgery, University Federico II, Naples, Italy.

[7] Medical Oncology, IRCCS-Sacro Cuore Don Calabria Hospital, Negrar di Valpolicella, Verona, Italy.

[8] Division of Medical Oncology, Department of Oncology and Hematology, University Hospital of Modena, Modena, Italy.

[9] Oncology Unit, ASST Papa Giovanni XXIII, Bergamo, Italy

[10] Medical Oncology, Università Cattolica del Sacro Cuore, Rome, Italy; Comprehensive Cancer Center, Fondazione Policlinico Universitario Agostino Gemelli, IRCCS Rome, Italy

[11] Gynaecological Oncology, Fondazione Policlinico Universitario Agostino Gemelli IRCCS, Rome, Italy

[12] Precision Medicine Unit in Senology, Fondazione Policlinico Universitario Agostino Gemelli IRCCS, Rome, Italy

***Correspondence:**
Dr. Patrizio Giacomini
Precision Medicine Unit in Senology
Fondazione Policlinico Universitario Agostino Gemelli IRCCS
Largo Agostino Gemelli, 8
00168 Roma
(Italy)

Running title: HER2-2D in advanced breast cancer

SUPPLEMENTARY METHODS

Cell lines

BT474, SK-BR-3, MDA-MB-231, and T47D were obtained as certified breast cancer cell lines from the American Type Culture Collection (ATCC). KPL-4 cells were from the originators [1]. All cells were grown in RPMI 1640/10% FBS (standard conditions). Genomic DNA (gDNA) was extracted by the SpinNAker Universal Genomic DNA mini kit (Euroclone, italy). For protein extraction, cells were lysed (approximately $1x10^7$ cells/ml) by 30 min incubation in CST lysis buffer supplemented with 1 mM PMSF and 0.1 mM aprotinin (Thermo Fisher Scientific). Lysates were centrifuged at 14,000 rpm (10 min), and protein concentration in the supernatant was assessed by the BCA Assay (QuantumProtein kit, Euroclone) prior to storage at -80°C in single-use aliquots.

Tumor tissues

Frozen tissue aliquots were also obtained through the IRE Institutional BioBank. Two distinct frozen tissue cylinders were cryostat cut, counterstained by hematoxylin/eosin, assessed for tumor fraction (from 20% to 90% of the cells) by an expert pathologist, and used to extract either gDNA (with the QIAmp DNA FFPE Tissue Kit; Qiagen, Hilden, Germany) or proteins, as follows. Tissues were homogenized at 60 mg/ml in CST lysis buffer supplemented with 1 mM PMSF and 0.1 mM aprotinin (Thermo Fisher Scientific) by a rotating blade homogenizer (Sorvall) for approximately 10 seconds, and then incubated for 30 min on ice. Lysates were cleared by centrifugation at 14,000 rpm (10 min), and protein concentration in the supernatant was assessed by the BCA Assay (QuantumProtein kit, Euroclone) prior to storage at -80°C in single-use aliquots.

dPCR assays

HER2F: ACAACCAAGTGAGGCAGGTC; HER2R: GTATTGTTCAGCGGGTCTCC; HER2 probe: FAM-CCCAGCTCTTTGAGGACAAC. EFTUD2F: GGTCTTGCCAGACACCAAAG, EFTUD2R: TGAGAGGACACACGCAAAAC, EFTUD2 probe: VIC-GGACATCCTTTGGCTTTTGA. dPCR conditions are automatically assigned by onboard QuantStudio 3D software (Thermo Fisher Scientific).

SUPPLEMENTARY RESULTS

dPCR and ELISA testing of breast cancer cell lines

As a first step to validate the two analytes of HER2-2D, gDNAs and soluble protein lysates were obtained from breast carcinoma cell lines known to be either HER2-amplified (BT474, SK-BR-3, and KPL-4) or HER2-neutral (T47D, MDA-MB231). HER2 DNA copy numbers and polypeptide levels were assessed by HER2/EFTUD2 dPCR, and a sandwich ELISA, respectively. As expected, HER2 DNA copies were ≥20 in HER2-amplified cells, and close to the unit in HER2-copy-number-neutral cells (Fig. S1a). As also expected, ELISA (Fig. S1b) detected similar levels of HER2 overexpression in HER2-amplified

cells, and either low or undetectable HER2 levels in HER2 DNA-copy-neutral cells (Fig. S1b). Lysates of HER2-neutral cells were deliberately loaded in higher amounts than those from HER2-amplified cells to reveal minute amounts of HER2 even in putative HER2-negative cells (Fig. s1b). However, quantitative differences were readily appreciated when equal lysate inputs (e.g. 0.5 μg) were compared (Fig. S1c). As expected, HER2-2D plots (dPCR vs ELISA) revealed a significant linear regression between HER2 DNA copy numbers and HER2 polypeptides in lysates (Fig. S1d), showing that dPCR and ELISA consistently estimate the HER2 DNA copy number/(over)expression dosage and the two measurements correlate.

dPCR and ELISA testing of breast cancer tumor tissues

Breast carcinoma tissues from untreated patients were divided in two aliquots. The former was formalin fixed and paraffin embedded, and then assessed for its HER2 status as per international ASCO-CAP diagnostic guidelines [2]. The latter was snap frozen and used to isolate genomic DNA and prepare tissue lysates. Lysates were serially diluted and tested by sandwich ELISA. An input of 0.5 μg total proteins per well (e.g. the same amount used for cell lysates) provided the greatest dynamic range to discriminate among different HER2 contents (Fig. S1e), and was selected as optimal. HER2 protein levels detected in tissue lysates were correlated with HER2 DNA copy numbers assessed by dPCR, resulting in a significant linear regression, as shown by the HER2-2D plot (Fig. S1f). In summary, HER2-2D detected a significant correlation between HER2 overexpression and amplification in tissues from untreated breast cancer patients, as expected.

HER2-2D in breast cancer plasma

Dilutions of blood plasma from healthy donors (n=4) and 4 breast cancer patients (HER2 subtype) were tested by ELISA to identify the dilution resulting in the greatest difference between putative-

negatives and putative-positives. The optimal resolution was obtained at the intermediate plasma input (used throughout in this report) of 1 μl (Fig. S1g).

aHER2 and sHER2 following treatment with Trastuzumab and Pertuzumab

cfDNA and plasma samples were obtained at progression from 32 patients who had received Trastuzumab (T) alone (n=10), or Trastuzumab plus Pertuzumab (T+P; n=22) for metastatic disease, with or without chemotherapy (Table 1). Testing for amplified HER2 (aHER2) and soluble HER2 polypeptides (sHER2) revealed sHER2 levels above the normal FDA threshold in a minority of patients progressing on T (4/10; 40%), and in an even lower percentage of patients progressing on T+P (3/22; 14%), with a significant difference in mean sHER2 levels (59.3 vs 11.0 ng/ml; two-tailed t test p= 0.03) between groups (Fig. S2a). In addition, HER2-2D plots (aHER2/sHER2; Fig. S2b) of the T group revealed a complete absence of single-positives, all patients (10/10; 100%) being either aHER2/sHER2 double-positives (n=4) or double-negatives (n=6). Similar to the T group, the T+P group displayed many double-negatives (18/22; 82%), but unlike the T group double-positives were entirely absent, and some aHER2 and sHER2 single-positives were seen (n=5 altogether; 23%).

These results confirm sHER2-high levels in some patients at progression from T [3], and in addition show that this occurs, but to a lesser extent, at progression from T+P. In addition, aHER2 and sHER2 are coordinated in all and most patients (100% and 77% respectively), minor aHER2/sHER2 dis-coordination being exclusively seen in a minority of patients receiving double-antibody blockade.

Baseline (T_0) sHER2 levels in blood do not correlate with PFS during T-DM1 treatment

Patients at T_0 were sorted into sHER2-low (n=20) and sHER2-high (n=17) based on sHER2 levels below and above the 15 ng/ml FDA-defined aHER2 threshold, respectively. In this classification

based on absolute, threshold-based, one-time pre-T-DM1 measurements, progression-free survival (PFS) was not significantly different between the sHER2-low and sHER2-high groups (Fig. S3a and b; p=0.34).

REFERENCES

1. Kurebayashi J, Otsuki T, Tang CK, Kurosumi M, Yamamoto S, Tanaka K, et al. Isolation and characterization of a new human breast cancer cell line, KPL-4, expressing the Erb B family receptors and interleukin-6. Br J Cancer. 1999;79(5-6):707-17.

2. Wolff AC, Hammond MEH, Allison KH, Harvey BE, Mangu PB, Bartlett JMS, et al. Human Epidermal Growth Factor Receptor 2 Testing in Breast Cancer: American Society of Clinical Oncology/College of American Pathologists Clinical Practice Guideline Focused Update. Arch Pathol Lab Med. 2018;142(11):1364-82.

3. Eppenberger-Castori S, Klingbiel D, Ruhstaller T, Dietrich D, Rufle DA, Rothgiesser K, et al. Plasma HER2ECD a promising test for patient prognosis and prediction of response in HER2 positive breast cancer: results of a randomized study - SAKK 22/99. BMC Cancer. 2020;20(1):114.

LEGENDS TO SUPPLEMENTARY FIGURES

Fig. S1. dPCR and ELISA validation in cultured cells, breast cancer tissues, and plasma samples. (a) dPCR-assessed HER2 DNA copy numbers (CN) in the indicated cell lines. CN=HER2/EFTUD2 ratio, calculated as follows: [n. of blue dots + n. of green dots]/ [n. of red dots + n. of green dots]. (b) HER2 polypeptide levels in soluble cell lysates tested by ELISA at 4 different dilutions. Lysates from HER2-low T47D and HER2-null MDA-MB-231 cells are less diluted, as noted. (c) HER2 polypeptide levels in BT474, SKBR3, KPL4, T47D and MDA-MB-231 lysates (same color as in panel b) at the same (0.5 μg) total protein input. (d) dPCR/ELISA results in panels (a) and (c) displayed by an HER2-2D plot: HER2 copy number in abscissa, HER2 proteins in ordinates. Regression is noted. (e) ELISA assessment of HER2 levels (OD readings) in 8 lysates from tumor tissues obtained at diagnosis, at 3 different dilutions, as indicated. Barplot ranges define the dynamic ranges of the ELISA assay, which are optimal at the intermediate total protein input. (f) HER2-2D (ELISA/dPCR) plot of copy number and HER2 polypeptide levels. Tumor DNA and lysates are from the tissues of patients #1 to #9. (g) HER2 ELISA dynamic ranges in the plasma of healthy blood donors (n=4) and patients with metastatic breast cancer of the HER2 subtype (n=4). Each plasma was tested at 3 different dilutions.

Fig. S2. HER2-2D in patients treated by naked antibodies. (a) 1d sHER2 plots in two patient subgroups treated with Trastuzumab (T) and Trastuzumab plus Pertuzumab (T+P), color-coded in green and orange respectively. (b) HER2-2D plot (aHER2 and sHER2 abscissae and ordinates, respectively) of the same patients, color-coded as in (a).

Fig. S3. sHER2 and survival. PFS of T-DM1-treated HER2-2D patient subsets with sHER2 levels above and below the FDA-approved (15 ng/ml) threshold at T_0 (sHER2-high vs sHER2-low). (a) dot

plots and (b) Kaplan-Meier analysis. Mann-Whitney and Mantel-Cox (log-rank) $p=0.34$, non-significant. Dotted red lines: median PFS, 182 days.

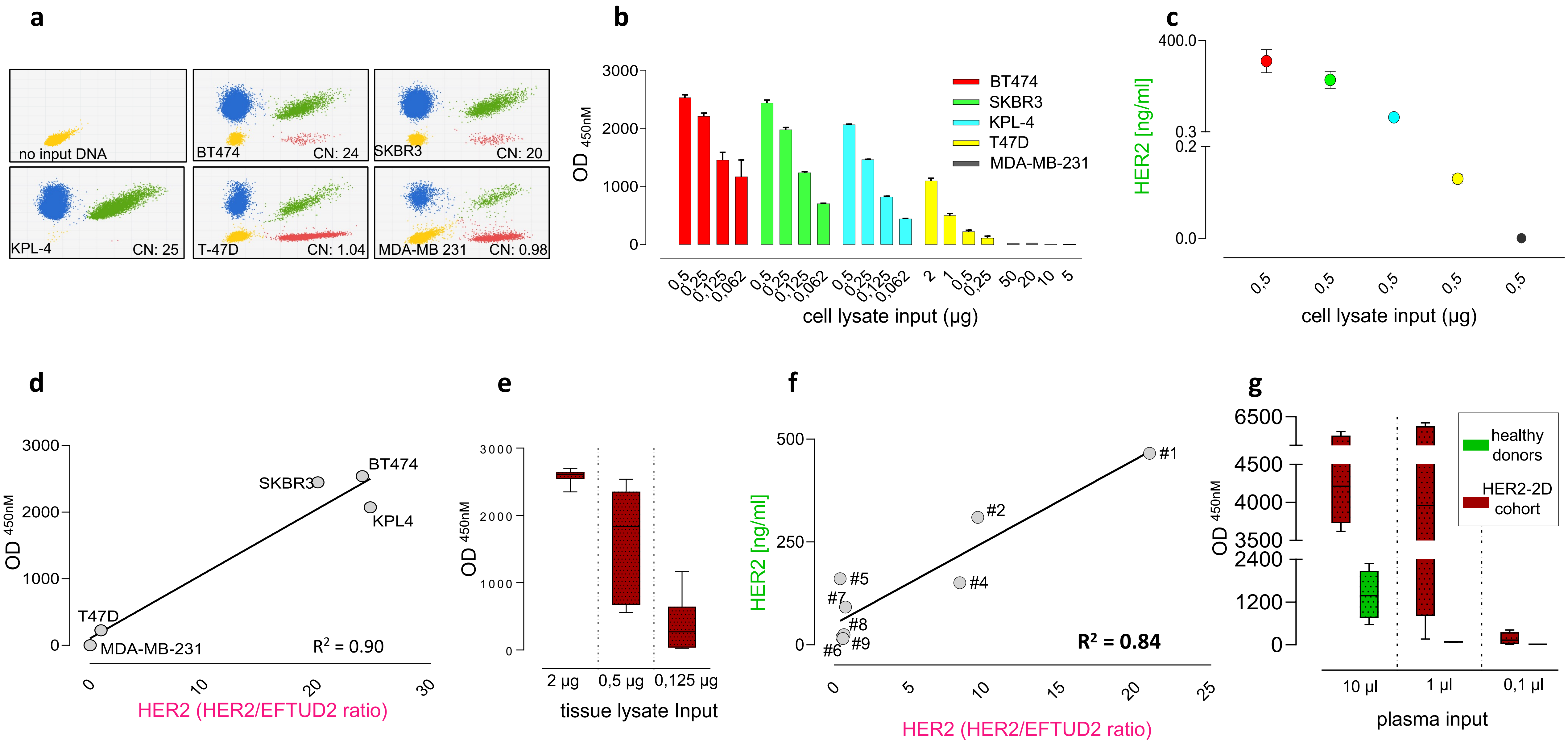

a
no input DNA
BT474
CN: 24
SKBR3
CN: 20
KPL-4
CN: 25
T-47D
CN: 1.04
MDA-MB 231
CN: 0.98
b
OD 450nM
BT474
SKBR3
KPL-4
T47D
MDA-MB-231
cell lysate input (µg)
c
HER2 [ng/ml]
cell lysate input (µg)
d
OD 450nM
BT474
SKBR3
KPL4
T47D
MDA-MB-231
R² = 0.90
HER2 (HER2/EFTUD2 ratio)
e
OD 450nM
2 µg
0,5 µg
0,125 µg
tissue lysate Input
f
HER2 [ng/ml]
R² = 0.84
HER2 (HER2/EFTUD2 ratio)
g
OD 450nM
healthy donors
HER2-2D cohort
10 µl
1 µl
0,1 µl
plasma input

Fig.S1

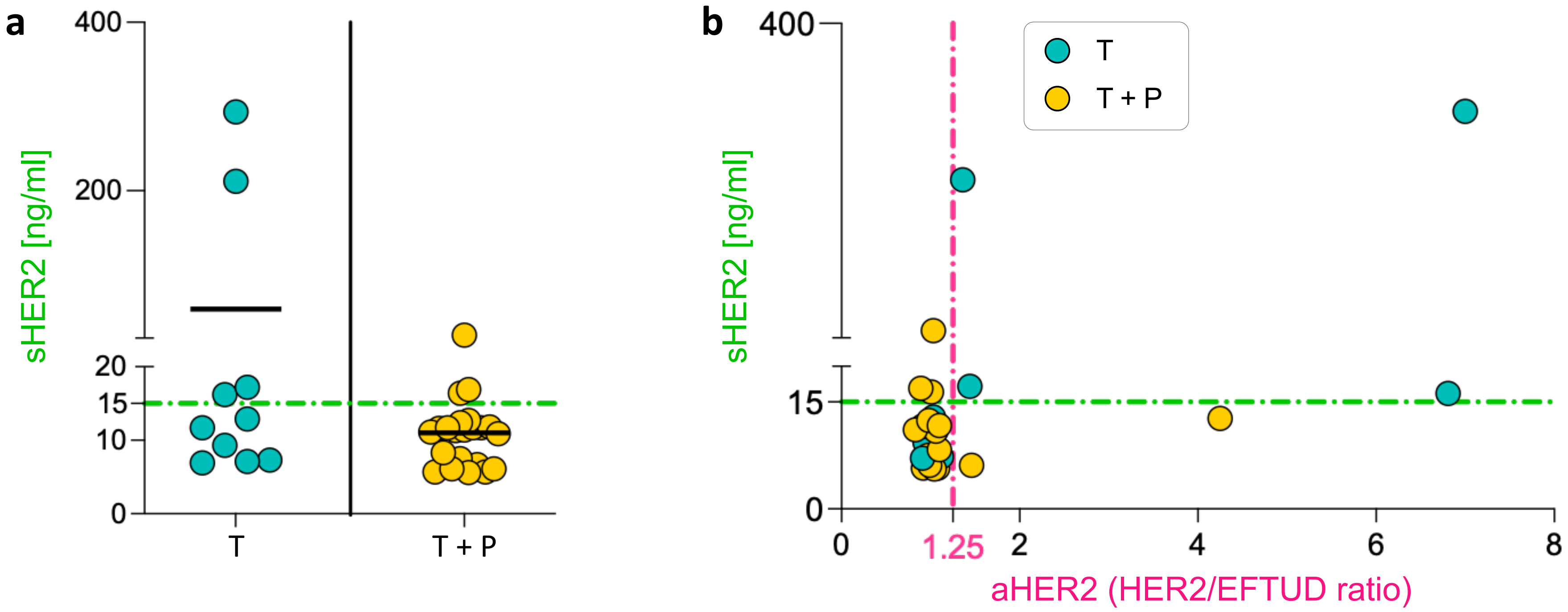
a
400
200
20
15
10
0
sHER2 [ng/ml]
T
T + P
b
400
15
0
sHER2 [ng/ml]
T
T + P
0
1.25
2
4
6
8
aHER2 (HER2/EFTUD ratio)

Fig.S2

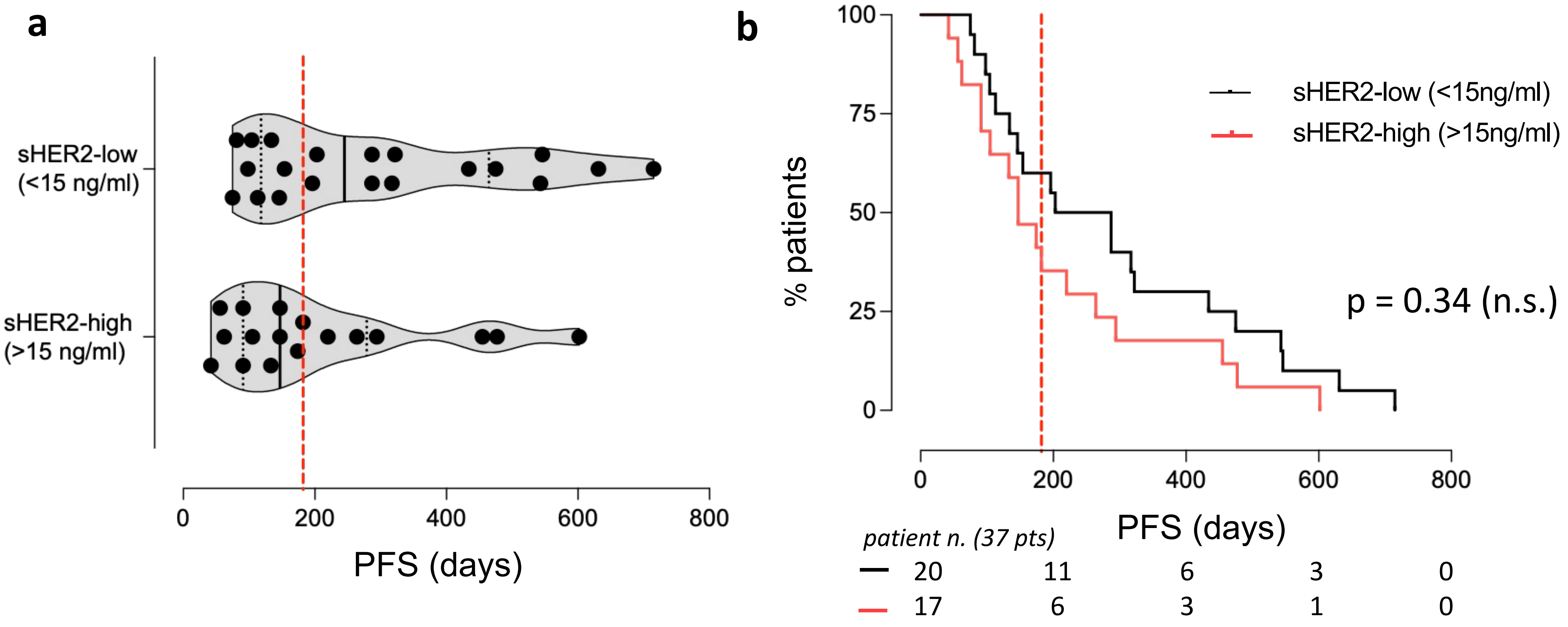

a
sHER2-low
(<15 ng/ml)
sHER2-high
(>15 ng/ml)
0
200
400
600
800
PFS (days)
b
100
75
50
25
0
% patients
sHER2-low (<15ng/ml)
sHER2-high (>15ng/ml)
p = 0.34 (n.s.)
0
200
400
600
800
PFS (days)
patient n. (37 pts)
20 11 6 3 0
17 6 3 1 0

Fig. S3
